# Characteristics associated with US Walk to School programs

**DOI:** 10.1186/1479-5868-4-67

**Published:** 2007-12-19

**Authors:** Dianne S Ward, Laura Linnan, Amber Vaughn, Brian Neelon, Sarah L Martin, Janet E Fulton

**Affiliations:** 1 Department of Nutrition, School of Public Health, University of North Carolina at Chapel Hill, North Carolina, USA; 2 Department of Health Behavior and Health Education, School of Public Health, University of North Carolina at Chapel Hill, North Carolina, USA; 3 Center for Health Promotion and Disease Prevention, University of North Carolina at Chapel Hill, North Carolina, USA; 4 Centers for Disease Control and Prevention, Atlanta, Georgia, USA

## Abstract

Participation in Walk to School (WTS) programs has grown substantially in the US since its inception; however, no attempt has been made to systematically describe program use or factors associated with implementation of environment/policy changes.

Describe the characteristics of schools' WTS programs by level of implementation.

Representatives from 450 schools from 42 states completed a survey about their WTS program's infrastructure and activities, and perceived impact on walking to school. Level of implementation was determined from a single question to which respondents reported participation in WTS Day only (*low*), WTS Day and additional programs (*medium*), or making policy/environmental change (*high*).

The final model showed number of community groups involved was positively associated with higher level of implementation (OR = 1.78, 95%CI = 1.44, 2.18), as was funding (OR = 1.56, 95%CI = 1.26, 1.92), years of participation (OR = 1.44, 95% CI = 1.23, 1.70), and use of a walkability assessment (OR = 3.22, 95%CI = 1.84, 5.64). Implementation level was modestly associated with increased walking (r = 0.18).

Strong community involvement, some funding, repeat participation, and environmental audits are associated with progms that adopt environmental/policy change, and seem to facilitate walking to school.

## Background

A recent literature review confirmed that physical activity (PA) is associated with important health benefits for children and adolescents, and recommended 60 minutes or more of moderate to vigorous PA daily [1]. This report adds to the growing body of literature recognizing the protective effects of PA against many chronic diseases including cardiovascular disease, diabetes, certain cancers, and osteoporosis for not only adults but children [2,3]. Additionally, the prevalence of overweight among children has more than doubled over the past 20 years [4] and declines in PA have been suggested as a cause of an energy imbalance driving the obesity epidemic [5].

Although researchers have not observed a significant decrease in leisure time PA [6,7], one component of PA that has measurably declined among American youth over the past 20 years is walking and bicycling to and from school. Between 1977 and 1995, active transportation to school (ATS) among children aged 5–15 years declined by 40% [8]. Although more recent transportation surveys reported a slight increase in the number of trips to school of 1 mile or less (31.3% in 1995 versus 35.9% in 2001) [9], current estimates still do not meet national health objectives [10]. For many children, especially those who live within 1 mile of school, ATS is a viable way of obtaining additional PA and has been shown to positively affect weight status [11].

In the United States, organized efforts to promote ATS, such as the Walk to School (WTS) program, emerged about 10 years ago and quickly spread from efforts in 2 cities in 1997 to all 50 states by 2002 [12]. WTS programs focus on promoting ATS through educational and promotional activities and by creating safe and supportive community climates for walking through policy and environmental change. Programs are typically anchored by the WTS Day, an international event that takes place the first week in October. Materials from the *iwalk *website describe how to implement a WTS Day event, activities that can extend the walking program throughout the year, and strategies for creating safe walking routes [13]. The WTS program's inclusion of both "downstream" (individual focused) and "upstream" (environmental/policy) approaches for promoting the use of ATS is consistent with the social ecologic model and supported by health behavior research [14-16]. The other major ATS initiative is Safe Routes to School (SRTS). Although WTS and SRTS have come to share similar goals, SRTS programs have traditionally focused primarily on pedestrian safety – using education, encouragement, enforcement, and engineering strategies to make it safer for children to walk to school.

Although two evaluations of Safe Routes to School (SRTS) programs in California examined the effect of programs with extensive education and traffic enforcement efforts [17] and engineering and infrastructure improvements [18]; characteristics associated with a broader application of WTS programs in the United States have not been formally assessed. Sponsoring WTS Day may introduce children and families to ATS; however, environment/policy efforts (e.g., adding a crossing guard) are thought to be more comprehensive, enduring, and impact a wider audience [16]. Knowing how the WTS program is used based on the complexity of its implementation (from promotion and education to policy/environmental change) and school and community factors associated implementation will provide a platform from which to develop effective WTS programming. Thus, this study examined school and community characteristics associated with level of implementation of WTS programs in the US.

## Methods

Using a cross-sectional approach, schools participating in WTS programs were surveyed about school and community characteristics; WTS activities, infrastructure, and resources; perceived level of program implementation; and perceived impact. A broad-based advisory panel (see Appendix) was convened to provide guidance on the evaluation process, including development of the initial survey. All procedures for the study were approved by the Institutional Review Board at the University of North Carolina at Chapel Hill.

### Sample

The study sample was recruited from the 2002 WTS registration database maintained by the Highway Safety Research Center (HSRC) at the University of North Carolina at Chapel Hill. This database, which allows schools to register their intent to participate in WTS Day activities [19], is the largest available source of WTS participants and has been maintained by HSRC since 2000. Individuals can register a single school, which is typically done by the local WTS coordinator, or multiple schools, which is typically done by the district WTS coordinator. It is not unusual for a district WTS coordinator to register 10 or more schools under one group registration.

The database used for recruitment in the present study included 782 WTS coordinators who represented individual schools, multiple schools, or school districts from all 50 states. Surveys were distributed in spring of 2003 and 605 were completed and returned. Of these, 9 were from schools not in the 2002 registration database, 27 were duplicates from the same school, 48 represented multiple schools or school districts in a single survey, and 71 were from schools that registered but did not participate in the 2002 WTS Day event. Therefore, 155 surveys were eliminated before analysis, resulting in a total of 450 surveys in the analytic sample.

### Survey measures

A preliminary survey was pilot tested with three active WTS coordinators to ascertain the relevance, clarity, and ease of survey administration. The final instrument is available from the primary author upon request. For this analysis, measures included: (1) *School demographics*. Six questions assessed the number of students enrolled, grade levels at the school, type of school (public, private, parochial), school organization (traditional, year-round, magnet, charter), percentage of students who qualified for free or reduced-price lunches, and percentage of students provided bus service. (2) *WTS program infrastructure and activities*. Seven questions assessed type of coordinator (local or district), assignment of WTS coordinator duties (to an employee, volunteer, etc.), years of participation in the WTS program, number and type of engaged community groups, amount of funding available that year, use of a walkability assessment, and other activities (e.g., additional walk days and promotional activities) offered in support of the WTS program. (3) *Level of program implementation*. Implementation level was determined by the school coordinator's response to the following question: *What activities or promotions did your school offer? *Possible responses were (a) participated in WTS Day, (b) participated in additional promotions outside of WTS Day, and (c) made policy or environmental changes to support ATS. Coordinators could select one or more of the responses to indicate their level of implementation. Programs were classified based on the "highest" level of participation selected: low (participated only in WTS Day), medium (participated in additional promotions), or high (made policy/environmental changes). *(4) Perceived impact*. Coordinators were asked:*In your opinion, in the past 12 months has there been an increase in the number of children who walk to school? *Responses included five levels, (*fewer*, *same*, *slight increase*, *moderate increase*, and *large increase*) which were collapsed into three levels for analysis (less, no change, and increase).

### Procedures

Each of the 782 coordinators in the 2002 WTS database were invited to participate by e-mail or regular mail depending on information provided in the registration database. E-mailed invitations included a link to an online survey; mailed invitations included a paper copy of the survey with a postage-paid return envelope. Those who did not respond within 3 weeks were contacted by telephone to complete the survey. These techniques are consistent with procedures designed to ensure a high response rate as described by Dillman [20]. Participants responded via Internet (n = 223, 49.6%), mail or fax (n = 107, 23.8%), and by telephone (n = 120, 26.7%).

### Data analysis

Frequencies, means, and standard deviations were calculated for each question, and schools were classified into 3 levels (low, medium, or high) on the basis of the single question about program implementation. Chi-square and *t *tests were used to compare survey responses across levels. In addition, Kendall's Tau-b statistic was used to determine the association between level of implementation and perceived impact of the WTS program on ATS since both variables are ordinal [21].

An exploratory proportional odds logistic regression model with a cumulative logit link was used to determine significant correlates of program implementation level. Nonsignificant variables were removed, one at a time, using a backward selection procedure at an alpha significance level of 0.05. To assess the relative importance of the remaining covariates, each was deleted, one at a time, from this reduced model, and change in log likelihood examined. Because the proportional odds assumption appeared to be violated for some covariates (p < 0.0001), a final "partial" proportional odds model [22], which assumes proportional odds for some covariates but not for others, was fit by using a general estimating equation (GEE) approach in SAS version 9.1.3, 2004 (SAS Institute Inc, Cary, NC), with PROC GENMOD.

## Results

### Univariate and bivariate analyses

#### School demographics (Table 1)

**Table 1:** Descriptive Characteristics of Schools by Level of Walk to School (WTS) Program Implementation, US Walk to School Evaluation Project, 2002^a^

	**WTS program implementation level**^**b**^			
		
	**Low (n = 251)**	**Medium (n = 105)**	**High (n = 94)**	**All (n= 450)**
Student Enrollment: Mean (SD)	549.3 (286.6)	531.1(251.3)	560.3 (236.1)	547.3 (268.1)

Grade Level: % (SE)^c^				
Elementary	87.6(2.1)	87.6(3.2)	87.0(3.5)	87.4(1.6)
Middle	8.8(1.8)	11.4(3.1)	13.0(3.4)	10.1(1.4)
High	3.6(1.2)	0.9(0.9)	1.0(1.1)	2.5(0.7)

School Type: % (SE)^c^				
Public	97.6(1.0)	97.1(1.6)	100(1.0)	98.0(0.7)
Private	0.4(0.4)	1.0(0.9)	0(-)	0.5 (0.3)
Parochial	2.0(0.9)	1.9(1.3)	0(-)	1.6(0.6)

School Type and Calendar: % (SE)^c^				
Traditional	91.1(1.8)	87.5(3.2)	89.1(3.2)	89.9(1.4)
Magnet	2.8(1.1)	1.9(1.3)	4.3(2.1)	2.9(0.8)
Charter	0.8(0.6)	1.9(1.3)	0(-)	0.9(0.4)
Year-Round (multi-track)	3.2(1.1)	2.9(1.6)	3.2(1.9)	3.2 (0.8)
Year-Round (modified)	2.0(0.9)	5.8(2.3)	3.2(1.9)	3.2 (0.8)

Percentage that Qualify for Free or Reduced-Cost Lunch: Mean (SD)^c^	43.0 (29.2)	45.3 (32.8)	47.3 (34.5)	44.4 (31.2)

Percentage Provided Bus Service: Mean (SD)^c^	32.2 (33.1)	31.6 (32.7)	29.2 (31.7)	31.4 (32.6)

a: No significant differences were found among levels for any of the characteristics listed.

b: Levels were defined as follows: low (participated in WTS Day only), medium (participated in promotions in addition to WTS Day), and high (made policy or environmental changes to support walking to school).

c: Percentages are based on the total responses to each question within a level.

Most (87.4%) surveys were completed for elementary schools. Average enrollment was 547.3 (± 268.1) students. Nearly all schools were public (98.0%) and organized around a traditional calendar (89.9%). On average, 44.4% of the students at these schools qualified for free or reduced-price lunch; 31.4% were eligible for bus service. To assess validity of responses, a 10% random sample of WTS surveys was selected and matched to archival data from state and national sources for enrollment and percent of students qualifying for free or reduced-price lunch (%FRPL). Within this sample, enrollment data were missing from 3, % FRPL data were missing from 12, and reference data could not be obtained for 1. With regards to enrollment, 61.0% of respondents estimated enrollment within ± 25 students and 80.5% within ± 50 students. With regards to percentage of students qualifying for free or reduced cost lunch, 65.6% estimated the percentage within ± 5% and 75.0% within ± 10%.

#### WTS program infrastructure and activities (Table 2)

**Table 2:** Walk to School (WTS) Program Characteristics and Activities Offered by Level of Program Implementation, US Walk to School Evaluation Project, 2002

	**WTS program implementation level**^**a**^				
			
	**Low (n = 251)**	**Medium (n = 105)**	**High (n = 94)**	**All (n= 450)**	**P value**
Years WTS offered^b^: % (SE)^c^					<0.0001
1 year	47.4 (3.2)	29.5 (4.5)	30.9 (4.8)	39.8 (2.3)	
2 years	24.7 (2.7)	25.7 (4.3)	20.2 (4.1)	24.0 (2.0)	
3 years	17.9 (2.4)	23.8 (4.2)	19.1 (4.1)	19.6 (1.9)	
4 years	6.4 (1.5)	16.2 (3.6)	20.2 (4.1)	11.6 (1.5)	
5 years or more	3.6 (1.2)	4.8 (2.1)	9.6 (3.0)	5.1 (1.0)	

Coordinator^b^: % (SE) ^c^					0.55
Local	88.8 (2.1)	92.1 (2.7)	85.2 (3.8)	88.8 (1.5)	
District	11.2 (2.1)	7.9 (2.7)	14.8 (3.8)	11.2 (1.5)	

Assignment of coordinator duties: % (SE) ^c^					0.003
Existing employee, no comp	37.4 (3.1)	40.8 (4.8)	37.2 (5.0)	38.2 (2.3)	
Existing employee, with comp		1.0 (1.0)	3.2 (1.8)	0.9 (0.5)	
Volunteer	30.5 (3.0)	38.8 (4.8)	36.2 (5.0)	33.6 (2.3)	
No official coordinator	32.1 (3.0)	19.4 (3.9)	23.4 (4.4)	27.3 (2.1)	

Number of Groups ^b^: % (SE) ^c^					<0.0001
1	15.8 (2.3)	3.9 (1.9)	9.7 (3.1)	11.8 (1.5)	
2–3	40.9 (3.1)	23.5 (4.2)	9.7 (3.1)	30.3 (2.2)	
4–5	24.7 (2.7)	18.6 (3.9)	23.7 (4.4)	23.1 (2.0)	
6+	18.6 (2.5)	53.9 (4.9)	57.0 (5.1)	34.8 (2.3)	

Money Spent^b^: % (SE) ^c^					<0.0001
None	40.2 (3.1)	16.8 (3.7)	18.7 (4.1)	30.4 (2.3)	
<$100	34.6 (3.0)	41.6 (4.9)	28.6 (4.7)	34.9 (2.1)	
$101–$500	19.1 (2.5)	30.7 (4.6)	33.0 (4.9)	24.7 (1.4)	
>$501	6.1 (1.5)	10.9 (3.1)	19.8 (4.2)	10.1 (2.4)	

Walk Assessment Conducted^b^: % (SE) ^c^	39.4 (3.1)	43.8 (4.8)	73.4 (4.6)	47.5 (2.4)	<0.0001

Activities Offered: % (SE)^c, d^					
Promotional activities^b^	63.7 (3.2)	79.0 (4.1)	86.2 (3.6)	72.4 (2.2)	<0.0001
Safety training^b^	39.9 (3.3)	58.0 (4.9)	57.4 (5.1)	48.2 (2.4)	<0.0001
Designated safe routes^b^	35.4 (3.2)	49.0 (5.0)	69.1 (4.8)	46.3 (2.4)	<0.0001
Adult-supervised walks^b^	21.5 (2.8)	46.0 (5.0)	51.1 (5.2)	34.1 (2.3)	<0.0001
WTS days throughout the year^b^	10.3 (2.0)	27.0 (4.4)	19.1 (4.1)	16.3 (1.8)	0.002
PE class activities	38.6 (3.3)	43.0 (5.0)	39.4 (5.0)	39.8 (2.4)	0.29
Academic activities^b^	23.3 (2.8)	39.0 (4.9)	37.2 (5.0)	30.2 (2.3)	0.0005
Other^b^	6.7 (1.7)	24.0 (4.3)	33.0 (4.9)	16.8 (1.8)	<0.0001

a: Levels were defined as follows: low (participated in WTS Day only), medium (participated in promotions in addition to WTS Day), and high (made policy or environmental changes to support walking to school).

b: *t *test, p < 0.05

c: Percentages are based on the total responses to each question.

d: Respondents were allowed to select more than one response to this question.

Overall, 88.8% of the respondents defined themselves as local WTS coordinators. Assignment of the WTS coordinator duties varied widely: 39.1% reported the WTS duties were incorporated into the responsibilities of an existing employee (less than 1% received additional compensation for these duties), 33.6% reported that duties were given to an unpaid volunteer, and 27.3% reported not having a designated person responsible for WTS. The majority (63.8%) had participated in the WTS program for 1–2 years. Over a third reported that 6 or more different groups were involved in their WTS efforts. WTS budgets ranged from none in 30.4% of schools to more than $500 in 10.1% of schools. When asked about specific WTS-related activities, the most commonly reported activities were poster campaigns and announcements (72.4%), pedestrian or bicycle safety training (48.2%), walkability or environmental assessments (47.5%), and designated safe routes for children's school travel (46.3%).

#### Level of program implementation

Two hundred fifty-one schools (55.8%) were classified as low implementation, 105 (23.3%) as medium implementation, and 94 (20.9%) as high implementation.

#### School characteristics and infrastructure by level of implementation

No significant differences in school demographics were found across the 3 levels of program implementation (Table 1). However, significant differences by implementation level were observed when assignment of coordinator duties, years of participation, number of community groups involved, and funding were considered (Table 2). For example, only 18.6% of low implementation schools reported 6 or more community groups involved in their efforts compared to 53.9% and 57.0% of medium and high implementation schools, respectively.

#### Type of activities by level of implementation

As noted in Table 2, significant differences were observed by level of WTS program implementation for all activities with the exception of those held within physical education classes. For example, 86.2% of high implementation schools engaged in promotional activities compared with 79.0% and 63.7% of medium and low implementation programs, respectively.

#### Perceived impact by level of implementation

Overall, 147 coordinators perceived at least some increase in the number of children using ATS. A modest, but significant, association between perceived increase in ATS and implementation level was observed (Kendall's tau-b = 0.186; 95% CI: 0.275, 0.961; p < .0001). In low implementation schools, only 27% noted an increase in the number of children using ATS compared to 35% of medium and 55% of high implementation schools, respectively.

### Multivariate analyses

To understand the significant independent correlates of WTS program implementation associated with level of implementation, we conducted an exploratory analysis with the following covariates: years of participation in the WTS program, number of groups involved in planning and implementation, use of a walkability assessment, funding, and two-way interactions between these variables. Nineteen schools (4.2%) were deleted because of missing covariates or outcomes assumed to be missing completely at random, leaving a sample size of 431 (low = 241, medium = 100, and high = 90). None of the 6 interaction terms was significant; therefore, the reduced model included the 4 main effect variables. Changes in log likelihood showed that that number of community groups involved had the most significant effect on the model, followed by funding, years of participation, and use of a walkability assessment (data not shown).

Cumulative logit plots of each covariate showed that the number of community groups involved and the use of a walkability assessment appeared to violate the proportional odds assumption of homogeneity among the ordinal scales of these two variables analyzed by level. Hence, for these two covariates, two separate odds ratios were used. The first compared the odds of being classified as low versus a higher level of program implementation (i.e., medium or high); the second compared the odds of being classified as high versus a lower level of program implementation (i.e., low or medium). For other covariates (years of participation and funding), proportional odds were assumed, so that the odds ratios were the same across all school level categories (Table 3).

**Table 3:** Adjusted Odds Ratios and 95% Confidence Limits for Levels of Walk to School (WTS) Program Implementation, US Walk to School Evaluation Project, 2002^a^

**Label**	**Estimate (SE)**	**Odds Ratio**^**c**^	**95% Confidence Limits**
Funding: Both Logits^b^	0.44 (0.11)	1.56	1.26, 1.92

Years of Participation: Both Logits	0.37 (0.08)	1.44	1.23, 1.70

Number of Groups Involved:			
(low vs. medium or high)	0.57 (0.11)	1.78	1.44, 2.18
(low or medium vs. high)	0.44 (0.13)	1.56	1.22, 1.99

Use of a Walkability Assessment:			
(low vs. medium or high)	0.78 (0.22)	2.17	1.42, 3.32
(low or medium vs. high)	1.17 (0.29)	3.22	1.84, 5.64

a: Levels were defined as follows: low (participated in WTS Day only), medium (participated in promotions in addition to WTS Day), and high (made policy or environmental changes to support walking to school).

b: The proportional odds model provides 2 response category comparisons: (1) school level low vs. medium and high collectively, and (2) levels low and medium vs. high.

c: Analyses were performed with control for the other 3 variables.

High level schools were significantly associated with more funding (p < 0.0001) and more years of participation (p < 0.0001). For every increase in funding category, the odds of being in a higher WTS implementation level increased by an estimated 56% (OR = 1.56, 95% CI = 1.26, 1.92). Likewise, for every additional year of WTS participation, the odds of being in a school with a higher implementation level increased by about 44% (OR = 1.44, 95% CI = 1.23, 1.70). For number of community groups involved and use of a walkability assessment, both of which appeared to violate the proportional odds assumption of homogeneity, results depended on response categories compared (OR 1 = low implementation schools compared to medium or high *versus *OR 2 = low or medium implementation schools compared to high). As the number of community groups involved increased, the odds of being a higher level school also increased. The effect is most dramatic when comparing low implementing schools to medium or high implementing schools. Each additional community group involved increases the odds of having a medium or high implementing school by 78% (OR 1 = 1.78, 95% CI = 1.44, 2.18). Conversely, each additional community group involved reduces the odds of being classified in the lowest level of program implementation by almost half (OR = 1 – 1/1.78 = 0.44). The effect is less dramatic as school level increases. For every additional community group involved, the odds of having the highest level of implementation increased by 56% (OR 2 = 1.56, 95% CI = 1.22, 1.99). On the other hand, the use of walkability assessments appeared to be most associated with highest level schools. Schools that completed an assessment were 3 times as likely to have made environmental/policy changes than schools that did not (OR 2 = 3.22), although the confidence interval was quite wide (95% CI = 1.84, 5.64). Again, these associations existed after adjustment for other covariates.

## Discussion

ATS can be an additional source of PA for school age youth [23-27]. Rates of PA participation, however, have declined over the past 20 years and, on average, less than 15% of schoolchildren regularly walk or bicycle to school [28]. Although organized efforts to promote ATS, such as National WTS Day, have been present since 1997 [12], this is the first article to describe WTS program implementation in the US and to examine how school and community characteristics are associated with level of program implementation.

Our findings indicate that use of the WTS program varies: more than one-half (56%) of the schools sampled implemented only a WTS Day event (low implementation), 23% implemented WTS Day and other activities (medium implementation), and 21% made policy/environmental changes to support walking to school (high implementation). Using multi-level modeling, we determined that number of community groups involved, amount of funding provided, years of participation, and use of a walkability assessment were significant, independent correlates of level of WTS program implementation. Additionally, we observed that programs making policy/environmental changes as part of their WTS program seemed to observe increased rates of ATS. These results have important implications for those who work with schools, communities, policymakers, and others to promote WTS programs in the US and may provide some direction for their work. Low implementation schools may be prime targets for intervention since they have demonstrated interest in ATS; however, more comprehensive programs should be encouraged to achieve maximum benefit from these efforts. This comparison of schools with different levels of program implementation has identified particular correlates that may guide intervention efforts to move low implementation schools toward more comprehensive programs.

Community involvement is an important factor associated with making environmental- or policy-related changes to support ATS. Our findings show that for every additional community group involved, the odds of having a more comprehensive WTS program increases by 56% to 78% (depending on response categories compared). Thus, having multiple community partners as part of a school-based program is likely to enhance the program's reach and engagement, especially in moving a program beyond the one day event. Among schools with high levels of WTS program implementation, 57% reported that 6 or more different community groups were engaged in their WTS efforts, specifically groups such as police, media, local businesses, members of planning commissions, parents, corporate sponsors, and elected officials. It is likely that the type and quality of these partnerships, as well as their number, are important for greater implementation. These findings are consistent with literature on the use of coalitions in health promotion activities. A recent review article on coalition effectiveness by Zakocs and Edwards reported that *membership diversity *was one of the six most commonly identified factors positively associated with coalition effectiveness [29]. Despite the challenges of organizing such a diverse effort, engaging a variety of partners appears to be critical to the long-term success of collaborative programs, including WTS.

Funding is another important independent correlate of level of WTS program implementation. Although it is not surprising that "money matters," our results indicate that the absolute amount of funding needed to mount WTS activities is fairly modest. Even among schools that made policy/environment changes, less than 20% had budgets greater than $500. It may be that it is not the money itself that matters, but the endorsement that a funded project brings to the groups working to increase ATS [30]. Resources, both direct and in-kind, are a fundamental component of successful partnerships and allow groups to create together more than could be produced alone [30]. These costs are not in the WTS budget but absorbed by the partnering agencies. For WTS programs, an annual budget of less than $500 may be adequate to create the synergy important in successful community partnerships [30]; however, more research is needed to understand the role of funding.

Length of involvement with the WTS program was also associated with higher levels of implementation. Almost half of the environment/policy group reported participation for three or more years compared to only 28% of the low implementation group. Although these data are cross-sectional and the cause-effect relation is unknown, we are encouraged to observe that experienced WTS Day participants are more likely to report activities that include additional programming and policy/environmental support. Sustainability of efforts to increase ATS is a key requirement for program success; however, maintaining such efforts is a major challenge [31]. Participation in the WTS program may be the first step toward the initiation of policy/environmental changes that support ATS. Prospective studies of community efforts to alter school travel patterns are necessary to fully answer this question [32].

Our findings also suggest that an environmental assessment is important as schools attempt to promote ATS. A walkability assessment, such as the Walkability Checklist [33], may help to identify areas around the school that need attention and may stimulate environmental/policy changes [34,35]. This type of assessment may increase partners' awareness of safety hazards and the need for change.

Although the WTS program can be used to promote a number of different objectives, many health advocates suggest ATS as a way to promote lifestyle PA. Therefore, the observed association between level of implementation and increased ATS, although modest (r = 0.18), is of great interest. Of those making policy/environment changes, more than half perceived increased ATS. However, it is promising to see that even among the schools conducting only the WTS Day event, more than 25% perceived change in ATS. Obviously, these assessments should be viewed with caution. WTS programs typically are unable to conduct formal impact evaluations and WTS coordinators may not be suitably objective as to the program's effect.

A sizeable number of surveys (n = 71) were eliminated from the analysis dataset because they failed to meet the inclusion criteria of participating in the 2002 WTS program. This group represents schools that thought they would participate, but failed to do so. Some had previously participated in WTS Day, while others were new to the program. Additional research is needed to elucidate why some schools that *could *participate in the WTS program do not.

Our study has several strengths. We: 1) used a credible source to identify a large number of WTS program participants, 2) assembled a national advisory panel to help us construct a survey instrument, and 3) employed a multivariate modeling analysis to identify important program characteristics. Along with these strengths, however, were some limitations. We report data only from coordinators listed in the 2002 WTS registration database who responded (60%) to the survey. We do not know how many other schools engaged in WTS activities during 2002, but did not officially register. However, the schools surveyed generally are not dissimilar from other US schools based on a number of characteristics. Using data from the National Center for Education Statistics (NCES), average school enrollment for 2002–2003 was 439 for elementary schools, 617 for middle schools, and 754 for high schools [36]. The majority (but not all) of our schools were elementary, so it is not surprising that the average school enrollment in this study (547) is more similar to that of elementary schools nationwide. Schools in this study also reported larger percentages of children qualifying for free or reduced price lunch (44.4% vs. 35.2%) and lower percentages of children being provided bus services (31.4% vs. 55.8%) compared to national data [36,37]. Lastly, program implementation was defined using a single question. Although validity of this question is unknown, it was developed with input from the advisory committee by using descriptions of programs described on the WTS website and the Kidswalk-to-School guide provided on the Centers for Disease Control and Prevention website [13].

Gaining a better understanding of characteristics and impact of WTS programs is needed, especially in light of the recent reauthorization of the Transportation Equity Act for the 21^st ^Century (SAFETEA-LU), which authorized $612 million in funding over the next 5 years for Safe Route To School (SRTS) programs in all 50 states. This bill allows states to hire a SRTS coordinator, and sets aside at least 10% (and up to 30%) of the funds for non-infrastructure activities that encourage ATS. Programs such as WTS Day and its associated activities appear to provide an important gateway event that schools can use to initiate, motivate, and organize community participation. Caution obviously must be observed when drawing conclusions based on cross-sectional data from a select group of WTS participants. However, these findings are useful for emphasizing some of the characteristics associated with more comprehensive WTS programs. Policy and environmental changes are thought to have an important impact on behavioral outcomes for many health behaviors [38], including ATS [39]. Public health officials and health advocates should recognize the importance of WTS activities as a low-cost, feasible approach to promote PA in youth.

## Competing interests

The author(s) declare that they have no competing interests.

## Authors' contributions

DW served as the project's PI providing oversight for study design, data analysis, and manuscript development. LL served as a Co-I providing assistance with the planning of the study design, data analysis, and manuscript development. AV served as project manager providing oversight of data collection and contributing to manuscript development. BN served as the project biostatician conducting data analysis, assisting with data interpretation, and contributing to the manuscript development. SM and JF served as project officers providing guidance on study design, data analysis, and manuscript development. All authors read and approved the final manuscript.

## Appendix

### Advisory Committee for Walk to School Project*

Marilyn Bull, Committee on Injury Prevention and Poison Prevention, American Academy of Pediatrics

Kathie Carothers, Public Safety, City of Chicago

Christina Economos, School of Nutrition Science and Policy, Tuffs University

Mark Fenton, walking consultant

Janet Fulton, Centers for Disease Control and Prevention

Karen Gerlach, Robert Wood Johnson Foundation

Derek Graham, Transportation Services, NC Department of Public Instruction

Mary Pat Hanley, National SAFE KIDS Campaign

Dorothea Hass, WalkBoston

William Hall, Highway Safety Research Center, UNC Chapel Hill

Chaun Hill, Transportation and Highway Safety, City of Phoenix

Wendi Kallins, Marin Safe Routes to Schools

Rich Killingsworth, Active Living By Design

Marilyn Leiber Governor's Council on Physical Fitness, Health and Sports

Lauren Marchetti, Pedestrian and Bicycle Information Center, UNC at Chapel Hill

Sarah Levin Martin, Centers for Disease Control and Prevention

Patrick McCormick, League of American Bicyclists

Tracy McMillan, Department of City and Regional Planning, University of California at Irvine

Barbara Quinn, St. John's District Pedestrian Awareness Project

Gwyn Robertson, Cumberland County Schools (NC)

Anne Seeley (deceased), University of California San Francisco

Jessica Shisler, Centers for Disease Control and Prevention

Bill Smith, Parent Representative

Cathy Thomas, Physical Activity and Nutrition Branch, NC Division of Public Health

Jeff Tsai, School Transportation Group, NC State University

Sally White, Palm Bay Elementary School (FL)

* Affiliations listed are those at the time of the study.
